# Cerebrovascular longitudinal atlas: Changes in cerebral arteries in unruptured intracranial aneurysm patients followed with MRA

**DOI:** 10.1016/j.nicl.2025.103766

**Published:** 2025-03-07

**Authors:** Aichi Chien, Fernando Vinuela, Viktor Szeder, Geoffrey Colby, Reza Jahan, Anthony Wang, Satoshi Tateshima, Gary Duckwiler, Noriko Salamon

**Affiliations:** a Div. of Interventional Neuroradiology, Dept. of Radiological Sciences, David Geffen School of Medicine at UCLA, Los Angeles, CA 90095, USA; b Magnetic Resonance Research Labs, Department of Radiological Sciences, David Geffen School of Medicine at UCLA, Los Angeles, CA 90095, USA; c Dept. of Neurosurgery, David Geffen School of Medicine at UCLA, Los Angeles, CA 90095, USA; d Div. of Neuroradiology, Dept. of Radiological Sciences, David Geffen School of Medicine at UCLA, Los Angeles, CA 90095, USA

**Keywords:** Cerebral arteries, Intracranial aneurysm, Machine learning, Longitudinal studies, Circle of Willis, Vascular remodeling, Aging

## Abstract

**Background:**

Patterns of change in cerebrovascular (CV) morphology associated with aging are highly relevant to the incidence and progression of CV disease, particularly stroke. Intracranial aneurysms (IA), a leading cause of hemorrhagic stroke, are linked with factors such as blood flow, arterial stiffness, and inflammation that may also drive other changes in CV morphology. We worked with a cohort of longitudinally-imaged IA patients to construct the first longitudinal atlas of CV morphology and studied its relationship with disease.

**Methods:**

110 IA patients, ranging from 19 to 84 years old at IA detection, were monitored using 3D magnetic resonance angiography (MRA) for a mean of 6.11 (2.60) years with 3.6 (1.3) scans per patient. Using 405 image studies, we applied a machine learning diffeomorphic shape analysis to construct a longitudinal atlas of the cerebral arteries which defined a general trajectory of CV morphological change vs. age. This was paired with a centerline analysis to verify changes in individual arteries.

**Results:**

Patient characteristics influenced the speed of CV shape change (e.g. diabetes mellitus, faster, p = 0.016), while other factors mapped to older CV age (e.g. hypertension, p = 0.0004). In parallel, we found that groups including autosomal dominant polycystic kidney disease (p = 0.0004), sex (p = 0.005), smoking (p = 0.046), and IA growth (p = 0.020) shared CV morphology characteristics. The centerline analysis validated changes consistent with the longitudinal atlas.

**Conclusion:**

A general CV trajectory of increasing artery length and tortuosity over a period of several decades was found. Although specific IA characteristics were not found to significantly affect this trajectory, these changes in the CV may contribute to increases in IA risk with aging. While our longitudinal findings were consistent with previous cross-sectional studies of individuals without IA, it remains to be determined whether the pattern of morphological change we observed is representative of aging within the general population. The model we developed provides a basis for integrating CV morphological change into understanding of aging and disease.

## Highlights

• From longitudinal MRA data, constructed a cerebrovascular longitudinal atlas using machine learning diffeomorphic shape analysis.
• Observed increases in cerebral artery length and tortuosity with age.
• Speed of arterial change and patient vascular age varied with disease.
• Patients with shared medical histories shared morphological characteristics.

## Graphical abstract

**Figure:**
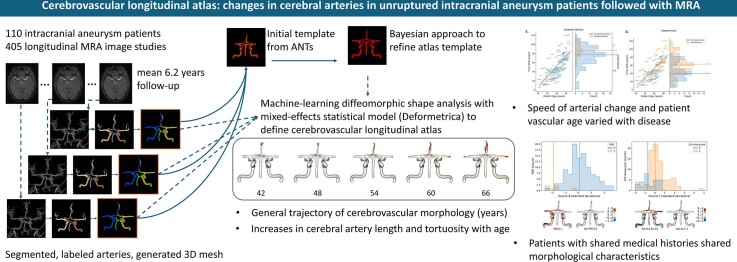

## 1 Introduction

The individual characteristics of patient cerebrovascular (CV) anatomy may contribute to various diseases, such as ischemic and hemorrhagic stroke (Chung et al., 2017, Deshpande et al., 2021, Manara et al., 2017; Fei-Fei et al., 2018). These characteristics are sometimes discussed as angioarchitecture, morphology, or anatomical differences. Large anatomical variations in the Circle of Willis (CoW), such as the absence of the anterior communicating artery (AComA) or posterior communicating arteries (PComA), may play a role in the incidence of ischemic stroke (De Caro et al., 2021, Jin et al., 2016, van Seeters et al., 2015). In addition, variations in cerebral artery shape and size, the focus of this study referred to here as CV morphology, have been associated with intracranial aneurysms (IA), a major cause of hemorrhagic stroke (Baharoglu et al., 2014, Chung et al., 2017, Duan et al., 2019, Tütüncü et al., 2014). Yet, CV morphology is not static. As part of normal development, CV morphology changes rapidly in the first years of childhood. As the brain grows, the diameters of cerebral arteries increase, accommodating increased blood flow requirements (Taylor et al., 2022, Wu et al., 2016). After childhood, studies suggest that CV morphology continues to change (Bullitt et al., 2010, Deshpande et al., 2022, Wright et al., 2013, Zhang et al., 2022). Previous studies have found arterial lengthening with aging, specifically within the basilar artery, and vertebral arteries, and internal carotid artery (Sun et al., 2022, Tanaka et al., 2013). These studies have generally been cross-sectional or measured one or a few specific arteries. As a result, age-related trends of CV morphology are not yet well understood. Of particular interest is CV morphology at and beyond middle age, as this age group is most impacted by CV disease. A lack of longitudinal data has so far made it difficult to confirm findings and define the trajectory of CV change with age.

In this context, patients with unruptured IA are an intriguing cohort because they are often followed with clinical neuroangiography over a period of years, providing a source of longitudinal images. Unruptured IA may be diagnosed when symptomatic or are sometimes incidentally detected with imaging. IA are relatively common among adults, with different studies estimating 2–5 % of the population harbors an unruptured IA (Vlak et al., 2011). Previous studies of patients with IA have associated CV morphology with local arterial hemodynamics that determine specific locations of stresses on the arterial wall implicated in IA formation and progression (Chien et al., 2018, Chung et al., 2017). Contributing factors to IA such as blood flow, arterial stiffness, and inflammation do change with aging (Roberts et al., 2023, Ungvari et al., 2018). As IA are a form of vascular remodeling, other changes in CV morphology might be expected to associate with IA. This study aimed to construct a more comprehensive model of CV morphology changes to better understand vascular aging and its potential relation with IA disease.

The majority of IA occur along the arteries that comprise the CoW (Brisman et al., 2006). The morphology of the CoW is highly variable across individuals. Specific arteries (internal carotid arteries (ICA), middle cerebral arteries (MCA), anterior cerebral arteries (ACA), etc.) must be present, but their shape and size vary significantly. Therefore, an individual’s longitudinal changes (changes which occur over time) in CV morphology must be somewhat specific to that individual. Previous cross-sectional studies have suggested there may be a shared pattern of CV morphological change (Bullitt et al., 2010, Deshpande et al., 2022, Wright et al., 2013, Zhang et al., 2022). Because of the need to disentangle variation over time from variation between individuals, we chose a mixed-effects statistical model to analyze 3D vascular shape at different time points for a group of individuals and synthesize the shared elements (Bône et al., 2020, Durrleman et al., 2014). Using images from a group of IA patients longitudinally followed with magnetic resonance angiography (MRA), we constructed a longitudinal atlas model of the cerebral arteries. In contrast to a standard vascular atlas, this model reconstructs the trajectory of CV change. In addition to this model, we made centerline-based measurements to identify longitudinal changes in specific cerebral arteries. With these approaches we addressed 1) whether there is a general, age-dependent trajectory of morphological change in the cerebral arteries of IA patients, and 2) whether certain demographics or groups with shared medical characteristics share common characteristics of CV morphology and change.

## 2 Methods

### 2.1 Data collection

This study was approved by the UCLA IRB. Patients were identified by reviewing clinical records and identifying patients with at least one unruptured IA with 3 years or more of MRA follow-up from 2013 January to 2023 December (252 patients). All patients had at least two MRA head image studies. Images were acquired using a 3D TOF MRA protocol on Siemens 3.0 T systems, typically with in-plane voxel size of 0.41 × 0.41 mm and a slice thickness of 0.64 mm (Nael et al., 2006, Villablanca et al., 2006). Images were evaluated for consistent image quality. Pediatric IA patients were excluded due to relative rarity, growth-related changes in cerebral arteries, and distinct IA etiology. When an increase in IA size was observed during follow-up, IA were treated under current guidance. Follow-up imaging after treatment was not included to exclude CV changes due to treatment. This yielded a set of 110 patients, 405 image studies for inclusion in this study. Electronic medical records for each patient were reviewed to record medical history and demographic information. In addition, MRA clinical imaging reports were reviewed to record IA-specific information. These data are summarized in Table 1. The 110 patients in this study had a total of 157 IA, located along the ICA, 110 IA; MCA, 22 IA; AComA, 20 IA; and posterior cerebral circulation (including posterior cerebral arteries (PCA), the basilar artery (BA), posterior inferior cerebellar arteries (PICA), and vertebral arteries (VA), 5 IA), reflecting location-dependent IA rates and likelihood of follow-up vs. treatment. The mean number of IA per patient was 1.4 (0.8), and mean initial size was 3.44 (1.68) mm. Over the longitudinal follow-up period of the study, the distribution of IA sizes did not significantly change: Kruskal-Wallis p-value = 0.392. The mean initial patient age was 57.32 (15.82) years, with a mean interval between the first and last included MRA image study of 6.11 (2.60) years, comprised of 3.6 (1.3) visits per patient.

**Table 1:** **Per-patient demographic and medical factors vs. longitudinal atlas acceleration and time shift values.** Group sizes indicate numbers of patients with a particular characteristic, such as an ICA aneurysm. Significant differences between groups from the longitudinal atlas results are indicated in bold. Acceleration indicates a difference in the speed of CV morphology change between the groups. Time shift indicates a difference in vascular age between the two groups. For race, group sizes were White: 56, Asian: 18, Black or African American: 2, More than one race: 3, and Unknown or Not reported: 31. For ethnicity, group sizes were Not Hispanic or Latino: 77, Hispanic or Latino: 10, and Unknown: 23. “Multiple aneurysm locations” indicates aneurysms in more than one of the four general locations (ICA, MCA, AComA, Posterior).

Group	Group sizes		Acceleration	Time Shift
	No	Yes	Kruskal-Wallis p value	Kruskal-Wallis p value
Male	91	19	0.284	0.185
Race (see table legend)			0.242	**0.022**
Ethnicity (see table legend)			0.247	**0.038**
Smoker	71	39	0.559	0.153
ADPKD	104	6	0.414	0.275
CKD	98	12	0.585	0.176
Hypothyroidism	82	28	0.190	0.778
Personal History of SAH	106	4	**0.023**	0.463
Family History of SAH	107	3	0.515	0.515
Seizure	101	9	0.723	0.715
TIA (Transient Ischemic Attack)	97	13	0.155	0.362
Stroke	90	20	0.566	0.932
Hypertension	49	61	0.169	**0.0004**
Dyslipidemia	45	65	0.059	0.431
Coronary artery disease	97	13	0.058	0.206
Atrial Fibrillation	99	11	0.672	0.665
Diabetes Mellitus	92	18	**0.016**	0.405
Thyroid diseases	70	40	0.619	0.610
Cancer	73	37	0.223	**0.034**
Other surgery	14	96	0.319	0.134
Alcohol	60	50	0.212	0.346
Stenosis of Carotid	90	20	0.510	0.337
Atherosclerosis/ICA Calcification	72	38	0.346	**0.001**
Family History of Aneurysm	95	15	0.221	0.354
Family History of Stroke	80	30	0.440	0.638
Head Trauma	107	3	0.551	0.354
Right Side aneurysm	48	62	0.123	0.819
Left Side aneurysm	57	53	0.818	0.855
Symmetric aneurysms	102	8	0.683	1.000
Multiple aneurysms	79	31	0.215	0.778
ICA aneurysm	27	83	0.126	**0.013**
MCA aneurysm	91	19	0.270	0.073
AComA aneurysm	90	20	0.141	0.846
Posterior aneurysm	105	5	0.824	0.971
Multiple aneurysm locations	95	15	0.152	0.705
Aneurysm growth	95	15	0.679	0.314

### 2.2 Data preparation

From the 3D MRA DICOM images, the major arteries comprising the CV circulation were segmented with a semi-automated process using 3D Slicer (Kikinis et al., 2014). For each image study, the corresponding clinical reports of IA size were used to calibrate the threshold to match the size of the segmented vessel with that of the recorded size. Local thresholding was used by the annotator, if necessary. Segmentation boundaries were the ICA: within C2 (Petrous), ACA: ∼5 mm past A2 bifurcation, MCA: ∼5 mm past M2 bifurcation, and for the posterior, BA, approximately the same axial plane as the ICA to ∼5 mm past the PCA P2 bifurcation. These boundaries were driven by practical concerns, specifically 1) inclusion of the arteries where most IA occur, 2) a preference for boundaries defined by arterial bifurcations or other anatomical features, and 3) a preference for boundaries which were consistently included in the MRA field of view, to maximize the inclusion of scans in the study. The additional artery segments beyond bifurcations were included so that preceding artery segments were more consistently segmented. When resolved in a particular image study, the smaller PComA and ophthalmic artery (OphA) were segmented along with the larger ICA, MCA, ACA, BA, and PCA. To assess intra-rater and inter-rater segmentation reliability, respectively, we compared image studies segmented twice by the same annotator (n = 5) and compared image studies segmented by different annotators (n = 5). We compared only the cerebral arteries used to generate the composite geometric models described in the next section. Intra-rater reliability was 0.97 (0.02) and 0.06 mm (0.05 mm) and inter-rater reliability was 0.91 (0.02) and 0.16 mm (0.03 mm) (Dice scores and average Hausdorff distances, mean and standard deviation, respectively).

Artery segments were manually labeled on the segmentation. Following labeling, we generated geometric mesh models of the CV arteries. Because the PComA is frequently either missing or not well resolved with MRA, we opted to split the cerebral arteries into three separate geometric models, rather than a single CoW model in which the missing arteries would increase topological variation between individuals. A series of Python scripts within 3D Slicer were used to generate composite models for the left anterior, right anterior, and posterior circulation from the segmentations for each image study. These scripts used the artery labels to combine artery segments and generated geometric meshes as inputs for the longitudinal atlas. For the anterior composites, the artery segments included: ICA, ACA A1, ACA A2, M1, AComA, for posterior: BA, PCA P1, and PCA P2. Arteries inconsistently captured with MRA, specifically the OphA and PcomA, were not included in these composite models. CV geometric variability increases significantly with each arterial branching. By including primarily more proximal artery segments, we limited the inter-subject CV variability to a level our longitudinal atlas could handle, while preserving the locations where the majority of IA occur.

Following generation of the three geometric models, a rigid registration was used to center and align the geometric models to the origin (Kikinis et al., 2014). For each image study, the orientation and distances between the three composite geometric models were maintained during this transformation. More precise registration is a central part of the longitudinal atlas described in the next section. In contrast, this pre-registration supported visualization and reduced the volume of space included for the initial atlas templates, improving performance. As this geometric data was being prepared as an input for the longitudinal atlas, a rigid registration, as opposed to elastic registration, was required to not interfere with the atlas modeling changes in CV morphology. After rigid registration, we used the Advanced Normalization Tools (ANTs) antsMultivariateTemplateConstruction2 method to generate the initial atlas templates from the set of patient CV models (Avants et al., 2011). These data preparation steps are depicted in Supplementary Fig. 1.

### 2.3 Longitudinal atlas creation

The longitudinal atlas used a mixed-effects statistical model that captures both the inter-subject variability in CV morphology between patients and the intra-subject change over time to create a multi-dimensional model of vascular change. We utilized the Deformetrica software package as a framework to create this model (Durrleman et al., 2014). Deformetrica uses a diffeomorphic approach for statistical analysis of 3D shape data. For the CV longitudinal atlas, it used machine learning to estimate a long-term scenario of shape changes from partially overlapping shorter-term individual observations. The data used were the three CV geometric models and patient age for each of the 405 image studies. The ANTs atlas provided the required starting point for the longitudinal atlas modeling, which substantially followed the methods of Koval and Bone et al. (Bône et al., 2020, Koval et al., 2021). To construct the longitudinal atlas, the three geometric models comprising the CV (left anterior, right anterior, and posterior) were present in the same space for a single statistical model. Preprocessing to initialize the model consisted of 1) generating high quality templates and calculating the noise standard deviation using a Bayesian approach, 2) generating individual geodesic regressions for the shape trajectory of each patient’s longitudinal vascular models, 3) using calculated deformation momenta shooting from average patient initial age to average age across image studies, 4) longitudinal registration, and 5) optimizing an initial longitudinal atlas using gradient ascent. This preprocessing completed after approximately two days. The resulting template, momenta, inertia, and other parameters were used to initialize the full longitudinal atlas model optimized using the MCMC-SAEM algorithm (Bône et al., 2018). This model took approximately 20 days to run on a 48-core AMD Epyc workstation with Nvidia RTX 3090 GPU. Using a base deformation kernel of 4 mm, the model was allowed 200 iterations to converge.

### 2.4 Longitudinal atlas interpretation

The atlas model encoded temporal and geometric variability with different parameters (Bône et al., 2020). The model calculated acceleration parameters to map each patient to the average CV shape progression, where acceleration values greater or less than the mean indicated, respectively, faster than average and slower than average speeds of CV morphological change. A reference time shift for the atlas was also determined, along with individual time shift parameters to map each patient to the average progression. Individual time shift parameters indicated patient vascular age, mapping each individual’s CV morphology against the atlas average.

The model encoded geometric variability using a set of 10 “sources” of geometric variation, where the atlas was defined at the mean of all the sources, and the variance was measured from the CV samples included in the model. The specified number of sources were generated by the Deformetrica software during the initialization process using independent component analysis. This function of Deformetrica is explained in detail by Bône et al., 2020 (Bône et al., 2020). The sources were modeled as independent samples from a normal distribution, yielding a continuum of deformations that stretched and twisted the space occupied by the CV arteries between extremes corresponding to the tails of the distribution. At the level of the samples, the weighting of the sources was relatively uniform. Because of the way the sources were generated, they were not limited to representing deformation in a distinct anatomical location. The CV geometry for a particular individual or the mean of a group of individuals could be unambiguously represented with a set of values for each of the 10 sources defining a composite deformation. These temporal and geometric parameters were used for statistical comparisons between patients grouped by shared demographics or medical history.

### 2.5 Arterial centerline measurements

Using 3D Slicer and the vascular modeling toolkit (vmtk), we measured the morphological characteristics of the arteries in each imaging study (Izzo et al., 2018). Measurements were obtained through an automated process of determining the centerline of each artery and calculating the length, tortuosity, and average radius along the length of the vessel. Tortuosity was calculated as the ratio of the length of the centerline to a straight line between the ends of the vessel. These measurement methods, as well as examples from two patients are shown in Supplementary Fig. 4. We then compared the values for each artery between the first and last image study for each patient. Patients without ACA A1 or PCA P1 segments were excluded from the ACA or PCA analyses (Table 2).

**Table 2:** **Comparison of artery centerline measurements between first and last image study for each patient.** Significant measures in bold. Measurements are summarized with medians and IQR. Radii are averages along the length of the artery. Prefix L- or R- indicates left or right. ACA combines A1 and A2 segments, PCA combines P1 and P2 segments, and MCA includes M1 segment. The general trend was an increase in vessel length and tortuosity, with significant changes found in several arteries. ACA radii notably decreased over the study interval. Sample numbers less than 110 indicate patients missing part of the artery (ACA A1 or PCA P1).

Variable	Samples	Initial	Final	Change	Percent Change	Wilcoxon p
RICA length (mm)	**110**	**77.48 (10.39)**	**78.59 (12.13)**	**1.02 (4.28)**	**1.38 (5.58)**	**0.002**
RICA tortuosity	**110**	**0.89 (0.25)**	**0.91 (0.28)**	**0.02 (0.08)**	**1.93 (9.12)**	**0.001**
RICA radius (mm)	110	2.05 (0.26)	2.06 (0.28)	0.02 (0.19)	0.93 (9.66)	0.231
RMCA length (mm)	**110**	**18.25 (10.65)**	**18.22 (9.73)**	**0.35 (2.18)**	**1.77 (11.44)**	**0.009**
RMCA tortuosity	110	0.08 (0.08)	0.08 (0.10)	0.00 (0.04)	2.17 (52.27)	0.373
RMCA radius (mm)	110	1.26 (0.20)	1.27 (0.18)	0.02 (0.18)	1.27 (13.72)	0.470
RACA length (mm)	104	47.58 (13.33)	48.63 (10.27)	0.32 (2.59)	0.85 (5.64)	0.195
RACA tortuosity	104	0.15 (0.12)	0.14 (0.10)	0.00 (0.04)	−1.64 (24.66)	0.270
RACA radius (mm)	**104**	**0.90 (0.25)**	**0.85 (0.20)**	**−0.04 (0.20)**	**−4.97 (21.05)**	**0.005**
LICA length (mm)	**110**	**78.24 (9.72)**	**78.62 (11.43)**	**0.82 (3.90)**	**0.92 (4.96)**	**0.006**
LICA tortuosity	110	0.86 (0.32)	0.89 (0.26)	0.00 (0.10)	0.02 (11.19)	0.522
LICA radius (mm)	**110**	**2.02 (0.28)**	**2.11 (0.28)**	**0.03 (0.16)**	**1.60 (7.65)**	**0.005**
LMCA length (mm)	**110**	**16.96 (9.62)**	**16.79 (10.33)**	**0.30 (2.59)**	**1.45 (13.84)**	**0.041**
LMCA tortuosity	110	0.08 (0.09)	0.08 (0.09)	0.01 (0.05)	7.49 (72.09)	0.209
LMCA radius (mm)	110	1.30 (0.23)	1.31 (0.18)	0.01 (0.20)	0.89 (15.08)	0.361
LACA length (mm)	**108**	**48.10 (11.86)**	**48.67 (14.36)**	**0.67 (2.48)**	**1.42 (5.43)**	**0.002**
LACA tortuosity	**108**	**0.12 (0.09)**	**0.14 (0.09)**	**0.01 (0.03)**	**6.42 (22.89)**	**0.0002**
LACA radius (mm)	**108**	**0.94 (0.22)**	**0.89 (0.21)**	**−0.03 (0.19)**	**−2.96 (19.62)**	**0.027**
BA length (mm)	110	29.58 (5.58)	29.87 (6.31)	0.35 (2.59)	1.33 (9.33)	0.215
BA tortuosity	110	0.10 (0.06)	0.10 (0.08)	0.00 (0.03)	2.52 (31.22)	0.980
BA radius (mm)	110	1.56 (0.25)	1.56 (0.29)	0.00 (0.16)	0.01 (10.51)	0.769
RPCA length (mm)	107	31.73 (10.73)	31.73 (11.23)	0.10 (3.02)	0.35 (9.35)	0.305
RPCA tortuosity	107	0.30 (0.20)	0.28 (0.29)	0.02 (0.11)	2.67 (38.04)	0.265
RPCA radius (mm)	107	0.94 (0.21)	0.91 (0.19)	−0.03 (0.17)	−2.76 (18.28)	0.150
LPCA length (mm)	108	33.09 (10.94)	32.08 (12.03)	−0.04 (3.53)	−0.09 (9.54)	0.951
LPCA tortuosity	108	0.31 (0.32)	0.26 (0.29)	0.00 (0.10)	−1.51 (34.17)	0.333
LPCA radius (mm)	108	0.94 (0.21)	0.94 (0.22)	0.01 (0.21)	1.27 (22.48)	0.870

### 2.6 Statistical methods

The Kruskal-Wallis test was used for comparisons between groups of patients, and the Wilcoxon signed-rank test was used to compare sample pairs of measurements taken at the beginning and end of the study interval. Descriptive statistics are presented as either “mean (standard deviation)” or “median (interquartile range)”, as indicated. The significance threshold was set at p < 0.05 for statistical testing. Post hoc tests were performed using the Holm-Bonferroni correction.

## 3 Results

### 3.1 Cerebral artery shape trajectory

**Fig. 1:**
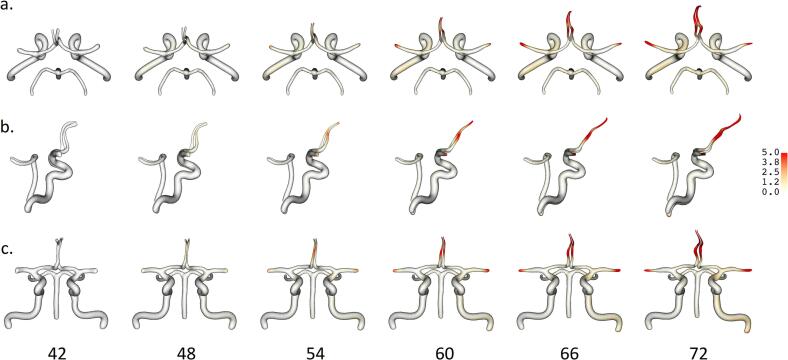
**Estimated population average trajectory over time for Circle of Willis arteries from longitudinal atlas model. a.** superior view, **b.** right view, **c.** anterior view. Bottom labels indicate vascular age in years. Color legend on right indicates displacement in mm relative to baseline vascular age model. Distances greater than 5.0 mm share same color. The model found a general vascular shape trajectory of increasing vessel length and tortuosity, most evident in the ACA and MCA.

**Fig. 2:**
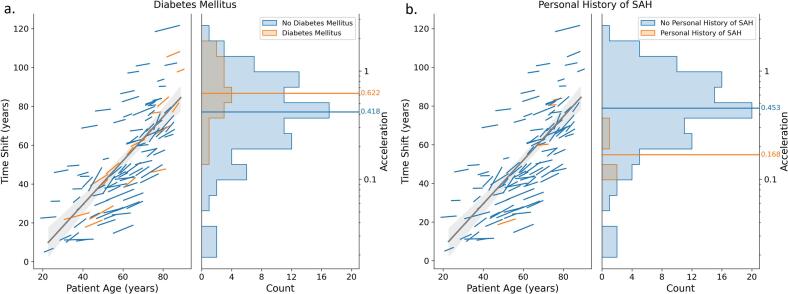
**a. Patients with Diabetes Mellitus showed a significantly higher acceleration (faster speed of vascular change, p = 0.016). b.** Patients with a history of a previous SAH showed a significantly slower speed of vascular change (p = 0.023). The left side of each plot present a line segment for each patient, colored according to group. The x-axis indicates the patient ages where follow-up began and ended. The midpoint of each line relative to the y-axis and the slope of the line respectively indicate the time shift (vascular age) and acceleration for that patient from the longitudinal atlas model. The grey line is a regression of time shift as a function of patient age. The right side of the plots present a histogram of acceleration values, along with lines indicating median accelerations. Larger values indicate a faster speed of CV morphology change. Note that acceleration is plotted on a log axis. Additional information is provided in Table 1 and Supplementary Table 1.

For the longitudinal atlas, the temporal variability of the shape trajectory across the included patients was described by a mean acceleration of 0.56 (0.44) and a reference time shift of 53.54 (24.01), less than the mean age across follow-up of 61.22 (15.32) years. These parameters are described in detail in Methods section 2.4. The general trend in CV morphology trajectory was towards longer, more tortuous arteries as age increased, evident in the ACA, MCA, and ICA (Fig. 1). A decrease in anterior cerebral artery (ACA) A2 caliber was also noticeable over time. To identify differences in CV shape change between groups, acceleration and time shift parameters were compared for a variety of medical history and demographic characteristics. These results are summarized in Table 1. Different factors were significant for acceleration and time shift. Because acceleration, the parameter describing the speed of CV change, is limited to positive values, its distribution was positively skewed. Patients with diabetes mellitus were found to have a significantly faster speed of vascular change (p = 0.016) (Fig. 2). Patients with a history of SAH had a significantly slower speed of change (p = 0.023).

**Fig. 3:**
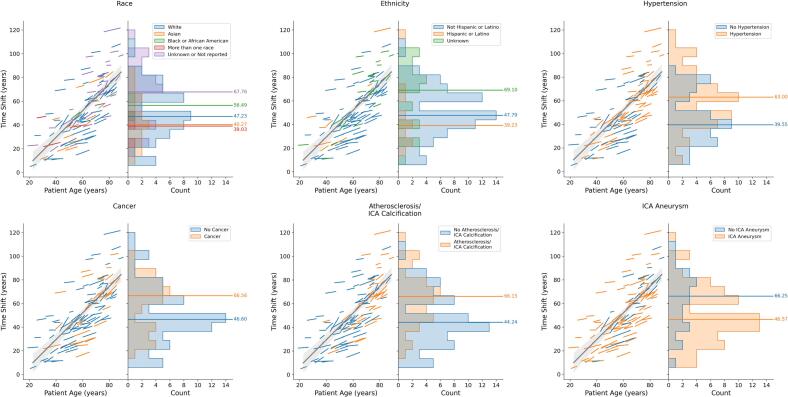
**Demographic and medical factors exhibit significantly different time shift (vascular age).** Time shift maps individual patients to overall longitudinal atlas trajectory of vascular change. Patients with hypertension (p = 0.0004), a history of cancer (p = 0.034), or atherosclerosis/ICA calcification (p = 0.001) showed significantly older time shift. Patients with ICA aneurysms showed significantly younger time shift (p = 0.013). As in Fig. 2, the left side of the plots present a line for each patient. The x-axis indicates the ages where follow-up began and ended. The midpoint of each line relative to the y-axis and the slope of the line respectively indicate the time shift and acceleration for that patient from the longitudinal atlas model. The grey line is a regression of time shift as a function of patient age. Histograms on the right side of plots share y-axis with left side. Horizontal lines indicate median time shift for groups. Larger values indicate older vascular ages. Additional information is provided in Table 1 and Supplementary Table 1.

Hypertension (p = 0.0004), cancer (p = 0.034), and atherosclerosis/ICA calcification (p = 0.001) were all associated with a later time shift, i.e. CV morphology mapping to a more advanced vascular age on the shape change trajectory (Fig. 3). Patients with at least one ICA aneurysm had a significantly earlier time shift (p = 0.013). Race was also found to be significant for time shift. Post hoc testing found the significant differences in time shift to exist between the Asian and “Unknown or not reported” categories (p = 0.037). While ethnicity was significant, post hoc testing did not establish a specific significant difference between the groups, with Unknown vs Hispanic or Latino p = 0.074. For both acceleration and time shift, Supplementary Table 1 provides the corresponding medians and interquartile ranges for the significant groups.

### 3.2 Association of patient medical history with arterial morphology

**Fig. 4:**
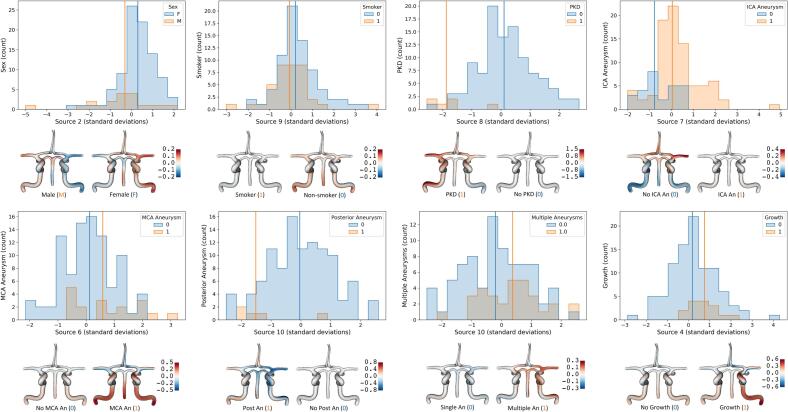
**Demographic, medical, and behavioral characteristics show differences in CV geometry.** Each group showed statistically significant differences in CV morphology which were captured by the indicated geometric source. Significance (p-values) are provided in Supplementary Table 2. Each subfigure presents a histogram for the two groups, along the axis of geometric variation described by the specific source. Vertical lines indicate group medians. Below each graph are CoW models relative to the mean atlas shape at center of the source (0 on the histogram plot). Each pair of CoW models shares a color bar, indicating the displacement of the model surface from the atlas (mm). Red shades indicate direction of deformation away from atlas model surface, while blue shades indicate deformation of vessel surface into atlas model. Morphological differences are described in the text. (For interpretation of the references to color in this figure legend, the reader is referred to the web version of this article.)

Shape differences between groups were compared using the set of geometric sources that represented the average shape of each group. These sources of geometric variation are described in Methods section 2.4. Comparing these values between groups of patients, we found specific groups that shared morphological characteristics. Among these examples, generally the larger group (for example, patients without autosomal dominant polycystic kidney disease (ADPKD), or patients without a posterior aneurysm) was closer to the mean shape, while the smaller group exhibited distinct differences. When the group numbers were more similar, such as patients separated by sex or by age, the two categories shape characteristics mapped in opposite directions from the mean shape of the source. Results comparing demographic and medical history against geometric variation are presented in Fig. 4 and Supplementary Table 2. ADPKD patients exhibited a significant difference in Source 8 (p = 0.0004), corresponding to a narrower ICA and a narrower, straighter ACA A2. Several groups were significant for Source 10, characterized by less distance between the left and right ICA, a longer MCA M1 and PCA, and a shorter ACA A2: Sex (p = 0.008), Multiple aneurysms (p = 0.041), Stenosis of Carotid (p = 0.041), and Posterior Aneurysm (p = 0.018). Two other IA locations were also associated with specific anatomical variations: ICA: Source 7 (p = 0.00041), longer, thinner PCA, more BA curvature, more tortuous ICA near OphA; MCA: Source 2 (p = 0.049) and Source 6 (p = 0.032), smaller, shorter ICA with a narrower bifurcation angle into ACA and MCA. We did not, however, find that any sources were significant for either left or right IA. CV morphology for aneurysms that grew during follow-up was also significant: Source 4 (p = 0.020), with a more tortuous ICA near the OphA and a narrower, straighter BA.

### 3.3 Centerline analysis of longitudinal arterial change

In addition to the longitudinal atlas model, we made complementary centerline measurements to directly compare the length, tortuosity, and average radius of individual cerebral arteries across patient image studies. The changes observed with the centerline measurements were consistent with those observed with the longitudinal atlas model (Fig. 1). Comparisons between paired image studies from the initial and final visit of each patient identified significant changes in the geometry of patient arteries over the study interval (Table 2). Several arteries showed an increase in length and tortuosity over time. Significant increases were observed in both right and left ICA length, right and left MCA M1 length, right ICA tortuosity, left ICA average radius, left ACA length, and left ACA tortuosity. In an exception to the trend of artery radius increase over time, the average radius of both the right and left ACA decreased over time, with more significant changes observed within the right ACA. While several individual patients exhibited large increases in tortuosity in the BA and left and right PCA over time, for the whole study group no significant changes were observed in the posterior arteries over the course of the study. Considering sex, smaller artery radii were found in females than males (respectively, right ICA: 2.02 (0.25) vs. 2.22 (0.13), p = 0.00001, right MCA: 1.25 (0.19) vs. 1.37 (0.16), p = 0.00026, left ICA: 1.99 (0.26) vs. 2.20 (0.23), p = 0.00003, left MCA: 1.28 (0.24) vs. 1.40 (0.16), p = 0.00086) (values in mm, median and IQR).

Finally, we compared the longitudinal atlas results with the centerline results. The artery segments of the longitudinal atlas at the ages shown in Fig. 1 were labeled. Length, radius, and tortuosity were then measured using the centerline approach described in the methods. Average values were then plotted using the Bland-Altman method (Supplementary Fig. 3). Because the atlas effectively calculates the average of the group, which we then measured, and the centerline approach measures individuals, which we then averaged, the two approaches are very different. However, values generally fell within 2 standard deviations on the plot, supporting similar findings for the methods.

## 4 Discussion

### 4.1 CV morphology trajectory

This study of CV morphology using MRA images collected from IA patients followed over time supports a progression of change in the CoW arteries with aging. The longitudinal atlas model we used allowed us to reconstruct decades of the trajectory from a set of patients individually followed for shorter lengths of time. The general trend observed was towards longer, more tortuous CV arteries as age increases (Fig. 1). Our longitudinal findings from IA patients generally confirm those of previous cross-sectional studies (Bullitt et al., 2010, Deshpande et al., 2022, Wright et al., 2013, Zhang et al., 2022). As with an earlier study of 100 healthy volunteers of ages ranging from 18-74 years, we also found a trend of larger vessel diameters in men, along with an increase in vessel tortuosity with age (Bullitt et al., 2010). Larger cerebral artery diameters in men generally correlate with larger intracranial volumes and have been documented by other studies in the past (Del Brutto et al., 2023). With the exception of the left ICA, we did not find a significant increase in vessel radius with age, but this may be due to a difference in methods, as we specifically compared longitudinal measurements in patients, as opposed to Bullitt et al. pooling single-timepoint measurements. Methodological differences may also explain the decrease in ACA radius we observed, as Bullitt et al. measured only the A1 segment of the ACA, whereas we also included A2.

Wright et al. also studied single-timepoint brain MRA images from 61 healthy volunteers ranging from 19-64 years, and similarly found increasing artery length and tortuosity with age (Wright et al., 2013). Their morphometric analyses included total length and fractal dimension to evaluate arteries beyond the CoW. Although this study included relatively few older adults, they did not find significant variations in arteries between genders or hemispheres of the brain, but did find that the lengths of different cerebral arteries tended to co-vary. Deshpande et al., in a more recent cross-sectional study, investigated aging, stroke and Alzheimer’s disease (Deshpande et al., 2022). Their healthy dataset was composed of the brain MRA collected by Bullitt et al., augmented with 66 healthy MRA scans from the OASIS-3 study (LaMontagne et al., 2019). Their general aging-related finding were similar, although they also performed additional analyses which found trends of increasing CV branching and complexity with age. Interestingly, among Alzheimer’s patients they found CV consistent with increased vascular age; unsurprisingly during acute ischemic stroke, MRA captured less extensive CV. The particularly large cross-sectional MRA study by Zhang et al. (1133 generally healthy participants) found increasing age to associate with decreased arterial density, along with increased radius and tortuosity (Zhang et al., 2022). This study focused on the M1 segment of the MCA and the BA. Their data suggest significant radius changes in middle-age (45–54), although this finding was complicated by differences in the sex ratio of participants between decades.

Much of the previous work on age-related changes in cerebral arteries has focused on the posterior circulation, specifically the BA. A 9-year longitudinal study involving 164 Japanese patients identified a possible correlation between the rate of BA radius change and cardiovascular events, both CV and coronary, with lower rates of BA change associating with such events (Takeuchi et al., 2019). Although previous studies have indicated an increased risk of IA rupture among Japanese patients, this study of the BA did not record IA (Greving et al., 2014). Another previous study identified racial differences in the relationship between BA diameter and length vs. age (Sudre et al., 2022). While we found some racial differences in the time shift of our longitudinal atlas model (Fig. 3, Table 1) more complete data collection would be necessary to interpret these differences. In another longitudinal study of the BA, Ngo et al. found that after an average of 10 years, the dominance of one branch of the VA tended to induce changes in the curvature of the BA (Ngo et al., 2020). Because IA follow-up focuses on the CoW, most of our images did not include the VA, so we were not able to perform a similar analysis. For our sample of cases, we did not find significant changes in the geometric characteristics of the BA or other posterior arteries (Table 2).

### 4.2 Association of demographics and disease with CV morphology trajectory

We found that certain subgroups sharing characteristics had significant differences in the speed and timing of CV morphology changes. Time shift, effectively a representation of vascular age, showed a correlation with patient age at the initial date of IA diagnosis (Supplementary Fig. 2). A similar correlation was observed in a previous study of progressive hippocampal atrophy in Alzheimer’s patients (Bône et al., 2018). However, while the majority of the medical factors we studied are more common with increasing age, only a few exhibited significant associations with vascular age in our model of CV aging (Table 1).

Our current longitudinal atlas results associate diabetes mellitus with an increased speed of CV change (Fig. 2). Diabetes is well documented to contribute to large vessel stiffening and endothelial abnormalities, and research suggests that it interacts with vascular aging processes that result in atherosclerotic plaque (Lakatta and Levy, 2003, Ungvari et al., 2020). Diabetes promotes vascular inflammation and reduces endothelial vasodilation (Assar et al., 2016, Ungvari et al., 2020). In addition to contributing to IA formation and progression, inflammation is also prominent in vascular aging, the effects of which are best understood in the larger arteries: increased arterial stiffness, dilation, with thickening of the intima and media (Wang et al., 2021). In combination, diabetes and aging have been observed to produce more severe vascular dysfunction and higher prevalence of vascular disease (Assar et al., 2016). Although the specific mechanisms in which aging and diabetes interact are not well understood, our finding of increased speed of CV change in IA patients is consistent with diabetes augmenting the effects of aging on the vasculature.

Our results also associated hypertension, atherosclerosis, and a history of cancer with more advanced vascular age (Fig. 3). Hypertension has been linked to an increased risk of IA rupture, as well as contributing to stroke and other CV diseases (Sonobe et al., 2010). Biologically, hypertension is associated with increased vascular stiffness, changes in vascular tone and atherosclerosis, and is additionally linked to a number of mechanisms of vascular aging, such as oxidative stress (AlGhatrif and Lakatta, 2015, Ungvari et al., 2020). Therefore, our finding that patients with these diseases have a significantly greater vascular age is consistent with existing clinical and mechanistic understanding of aging. Taken together, these results support aging-related changes in CoW morphology, modulated by a variety of medical conditions.

### 4.3 Association between disease and CV morphology

The longitudinal atlas model we presented combined ten geometric source modes to represent the CoW in individual cases. Whereas a clinician would specifically recognize a localized dilation in the ICA as an IA, each source of variation in the model includes multiple deformations in space, allowing the current model to represent the overall shape and changes of the arteries that comprise the CoW. The application of diffeomorphic shape analysis to CV change and disease is new. To date, arterial morphology studies of IA have generally focused on the local area around the IA, most often the MCA bifurcation. Although in the current study we did not specifically investigate bifurcation angles, they have in the past been indicated as a contributing factor to IA (Baharoglu et al., 2014, Miyata et al., 2023, Tütüncü et al., 2014). Arterial characteristics such as bifurcation angles are implicitly incorporated into the CV longitudinal atlas by the inclusion of the arteries in the model. Past longitudinal studies of IA related morphological changes have similarly been focused on changes to the IA itself, particularly growth (Chien et al., 2019, Miyata et al., 2023, Ramachandran et al., 2015). We are not aware of research examining IA related longitudinal changes in the CV.

Several demographic and medical history factors were found to correlate with one or more source of geometric variation (Fig. 4, Supplementary Table 2). These findings indicate that general CV shapes that associate with specific diseases may be identified. ADPKD is well-documented to manifest with intracranial dolichoectasia, as well as IA (Perrone et al., 2015). Similarly, in past studies of Fabry disease, BA length and tortuosity were both found to correlate with age in both patients and controls, with differences in radius observed between male patients and controls (Manara et al., 2017). Our findings also indicate that morphological variations may contribute to IA in specific locations, as well as continued IA growth. By quantifying vascular characteristics of the system of CV arteries, it may be possible to create new vascular shape biomarkers to diagnose and track disease that complement existing indicators.

The dataset of longitudinally followed IA patients allowed this study to investigate associations between IA characteristics and the trajectory of CV change. Previous studies have associated specific CV morphological characteristics, such as more tortuous arteries, with IA occurrence and development (Baharoglu et al., 2014, Chung et al., 2017, Kliś et al., 2019). Therefore, changes in CV morphology that occur with aging may contribute to rates of IA alongside other risk factors. Currently, IA are difficult to predict, with the vast majority remaining undiagnosed prior to rupture (Rinkel et al., 1998). IA risk is multi-factorial, and CV morphology combined with vascular mechanical and cellular changes associated with aging likely influence their formation. While there are familial risks, the genetic basis for these remains poorly understood, and sporadic IA are more common (Bakker and Ruigrok, 2021). Placing IA within the context of overall CV change may help identify better predictors of IA risk. Our inclusion criteria accepted patients with a history of SAH if they had another untreated IA; while this was associated with a slower speed of vascular change, we are unable to draw conclusions because there were only 4 such patients in this study. It is not clear what mechanism could produce the observed difference in patients with a history of SAH. Notably, we did not find a difference in the rate of CV morphological change between patients with and without IA growth over the period of follow-up.

### 4.4 Limitations

Although it is implicitly part of the longitudinal atlas, our study did not explicitly investigate the contribution of anatomical variants in the CoW found in different individuals. The number of cerebral artery variants have been recognized for many years (Kayembe et al., 1984, Windle, 1888). A few patients included in this study did lack one of the ACA A1 or PCA P1 segments, thus the reduced number of measurements in those categories in Table 2. But, to identify a general trend of CV change, the current study focused on the common features shared between CoW. We did not include the PComA, which, when it is smaller, is not always consistently imaged with MRA. Likewise, vasodilation and other short-term processes may affect the images being captured, although within this sample of IA patients, images were captured under clinical, non-emergency settings, with multiple image studies included for each patient. Although IA are more prevalent among women (2/3 to 3/4 of IA patients), the higher proportion of female patients in this study may indicate more women returning for IA monitoring with MRA (Vlak et al., 2011). In this study, mean follow-up duration was 6.11 (2.60) years. Longer follow-up duration and more scans would improve the ability to observe changes in CV morphology within individuals. A longitudinal study which combined morphology with cerebral hemodynamics could provide additional context for the pattern of CV changes observed (Chien and Viñuela, 2017, Coccarelli et al., 2024). Additional study is needed to clarify whether the results apply to the population of healthy individuals, or to what extent the morphological changes over time were related to IA. Because MRA is uncommon among healthy individuals in the United States, this would require a prospective study of long duration.

## 5 Conclusion

We studied the morphological changes in a group of IA patients longitudinally-imaged with MRA and constructed a longitudinal atlas model of CV morphology based on a machine learning approach. Patient characteristics were found to associate with speed and extent of CV change. Because specific morphology characteristics have previously been associated with IA progression, the increases in arterial length and tortuosity we observed may contribute to the development of IA later in life. Current understanding of changes in CV morphology is limited. This study can provide a basis for future clinical investigation of CV morphology and the changes it may undergo over time in response to normal aging and disease.

## CRediT authorship contribution statement

**Aichi Chien:** Writing – review & editing, Writing – original draft, Visualization, Validation, Supervision, Software, Resources, Project administration, Methodology, Investigation, Funding acquisition, Formal analysis, Data curation, Conceptualization. **Fernando Vinuela:** Writing – review & editing, Supervision, Resources, Project administration, Methodology, Investigation, Data curation, Conceptualization. **Viktor Szeder:** Writing – review & editing, Supervision, Resources, Project administration, Methodology, Investigation, Data curation, Conceptualization. **Geoffrey Colby:** Writing – review & editing, Supervision, Resources, Project administration, Methodology, Investigation, Data curation, Conceptualization. **Reza Jahan:** Writing – review & editing, Supervision, Resources, Project administration, Methodology, Investigation, Data curation, Conceptualization. **Anthony Wang:** Writing – review & editing, Supervision, Resources, Project administration, Methodology, Investigation, Data curation, Conceptualization. **Satoshi Tateshima:** Supervision, Resources, Investigation, Data curation, Conceptualization. **Gary Duckwiler:** Writing – review & editing, Supervision, Resources, Project administration, Methodology, Investigation, Data curation, Conceptualization. **Noriko Salamon:** Writing – review & editing, Supervision, Resources, Project administration, Methodology, Investigation, Data curation, Conceptualization.

## Funding

This study is supported in part by NIH R01HL152270.

## Declaration of competing interest

The authors declare that they have no known competing financial interests or personal relationships that could have appeared to influence the work reported in this paper.

## Data Availability

Imaging data for this study is not publicly available to protect the privacy of participants. The code is available to the research community from the corresponding authors upon reasonable request.

## Appendix A Supplementary data

Supplementary Data 1 Data preparation pipeline, anterior view of Circle of Willis arteries. a. Example patient MRA image study. b. Arterial segmentation, with labels provided for the major arteries of the patient’s right-side. For the current study, ACA includes both A1 and A2 segments, and PCA includes P1 and P2. MCA includes only the M1 segment. c. Color-coded separate anterior and posterior lumen surface-mesh models. d. ANTs-derived initial template based on all individuals. e. Refined atlas template from Deformetrica pre-processing.

Supplementary Data 2 Linear regression comparing individual patient ages at initial aneurysm detection and individual time shift parameters from longitudinal atlas model. Both axes are plotted in units of years. Time shift (vascular age) correlates with detection age (patient age at the time of the first MRA image study). R2 = 0.535, p << 0.00001.

Supplementary Data 3 Bland-Altman comparison of longitudinal atlas vs. centerline measurements. Longitudinal change values for individual cerebral arteries are labeled. a. Length and b. radius are in units of mm. c. No units for tortuosity. Regression line for all arteries is plotted in gray. For the longitudinal atlas, the average CV morphology is first determined, and then the arteries of this shape are measured. For the centerline-analysis, individual arteries are measured first, and then the measurements are averaged. Although the two methods of measuring longitudinal shape change are very different, values generally fell within 2 standard deviations on the Bland-Altman plot.

Supplementary Data 4 Calculation of centerline measures and comparison of ICA tortuosity between two individuals. a. Characterization of left ICA length, radius, and tortuosity. Left ICA tortuosity = 0.88. b. left ICA segment from second patient, tortuosity = 1.27.
