# Supplementary figures and images for: Cerebrovascular longitudinal atlas: Changes in cerebral arteries in unruptured intracranial aneurysm patients followed with MRA
Source: Neuroimage Clin. 2025 Mar 7;46:103766. doi: 10.1016/j.nicl.2025.103766 (PMC11960659; doi:10.1016/j.nicl.2025.103766)

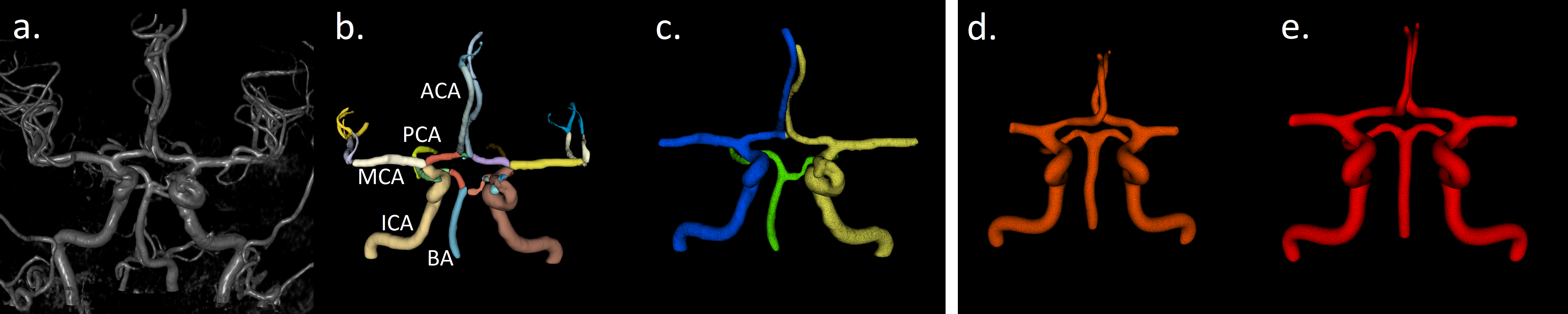

Supplement: Supplementary Data 1 — Data preparation pipeline, anterior view of Circle of Willis arteries. a. Example patient MRA image study. b. Arterial segmentation, with labels provided for the major arteries of the patient’s right-side. For the current study, ACA includes both A1 and A2 segments, and PCA includes P1 and P2. MCA includes only the M1 segment. c. Color-coded separate anterior and posterior lumen surface-mesh models. d. ANTs-derived initial template based on all individuals. e. Refined atlas template from Deformetrica pre-processing. [file mmc1.zip › Supplementary_Figure_1.png]

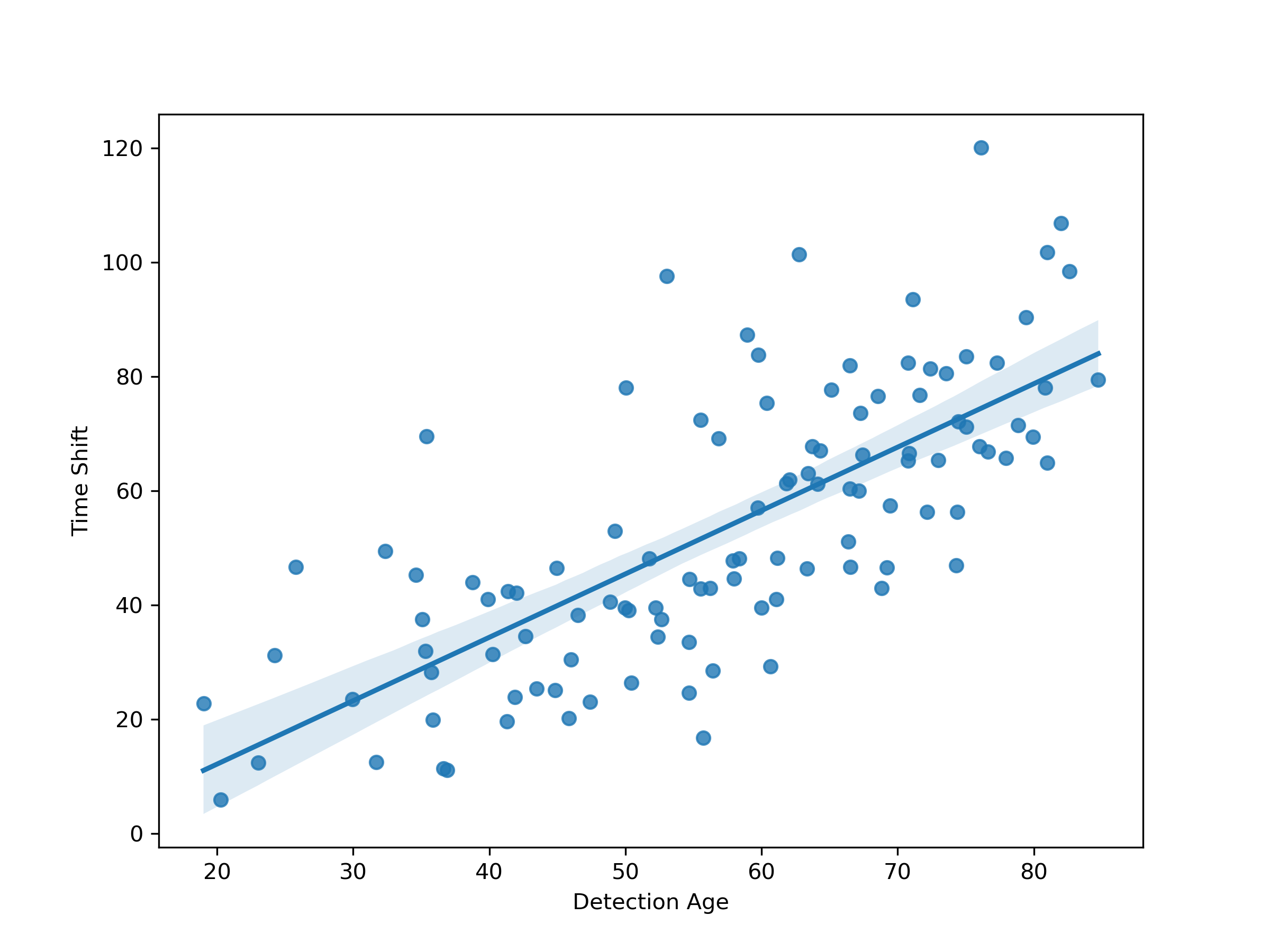

Supplement: Supplementary Data 2 — Linear regression comparing individual patient ages at initial aneurysm detection and individual time shift parameters from longitudinal atlas model. Both axes are plotted in units of years. Time shift (vascular age) correlates with detection age (patient age at the time of the first MRA image study). R2 = 0.535, p << 0.00001. [file mmc2.zip › Supplementary_Figure_2.png]

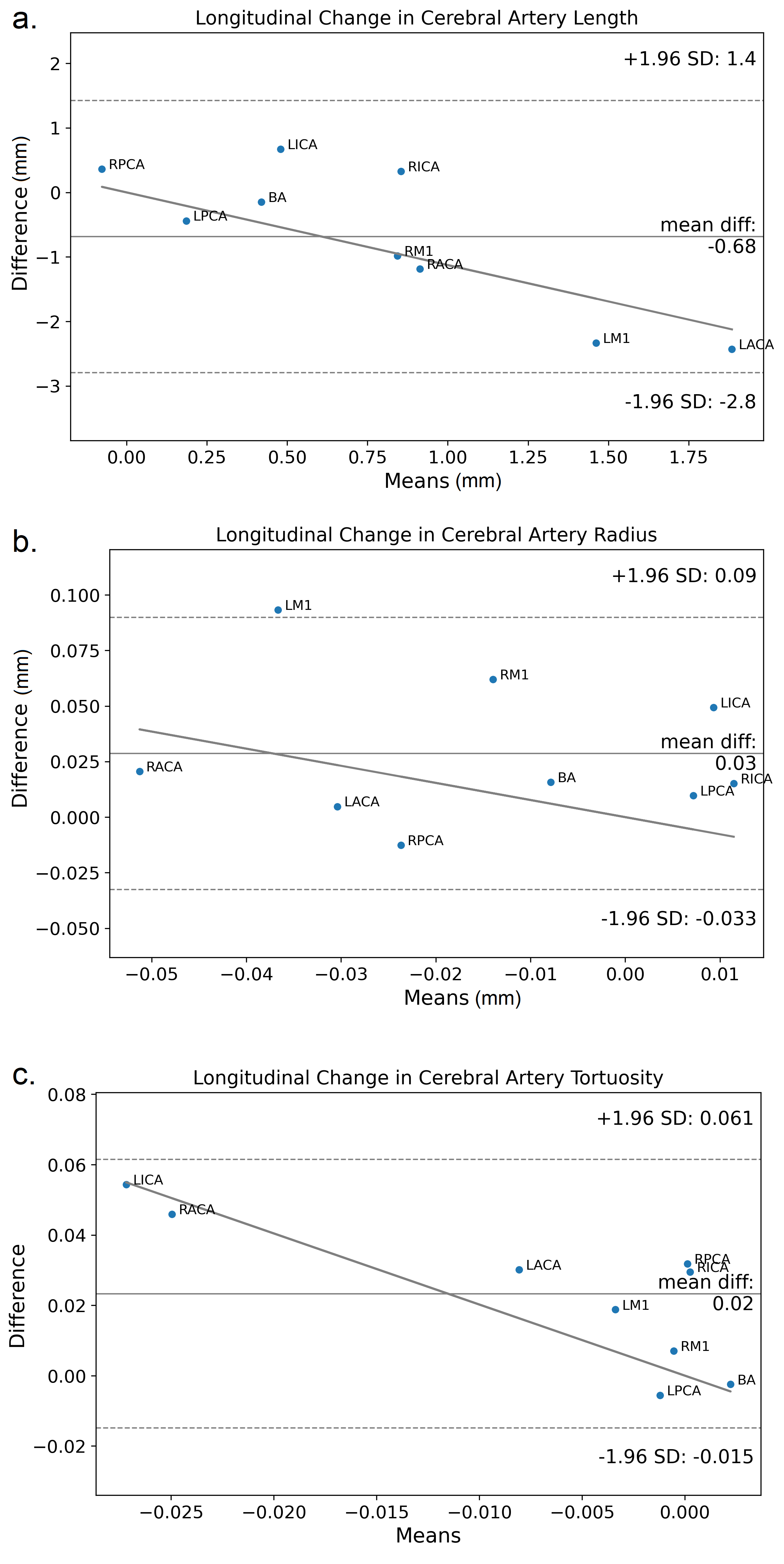

Supplement: Supplementary Data 3 — Bland-Altman comparison of longitudinal atlas vs. centerline measurements. Longitudinal change values for individual cerebral arteries are labeled. a. Length and b. radius are in units of mm. c. No units for tortuosity. Regression line for all arteries is plotted in gray. For the longitudinal atlas, the average CV morphology is first determined, and then the arteries of this shape are measured. For the centerline-analysis, individual arteries are measured first, and then the measurements are averaged. Although the two methods of measuring longitudinal shape change are very different, values generally fell within 2 standard deviations on the Bland-Altman plot. [file mmc3.zip › Supplementary_Figure_3.png]

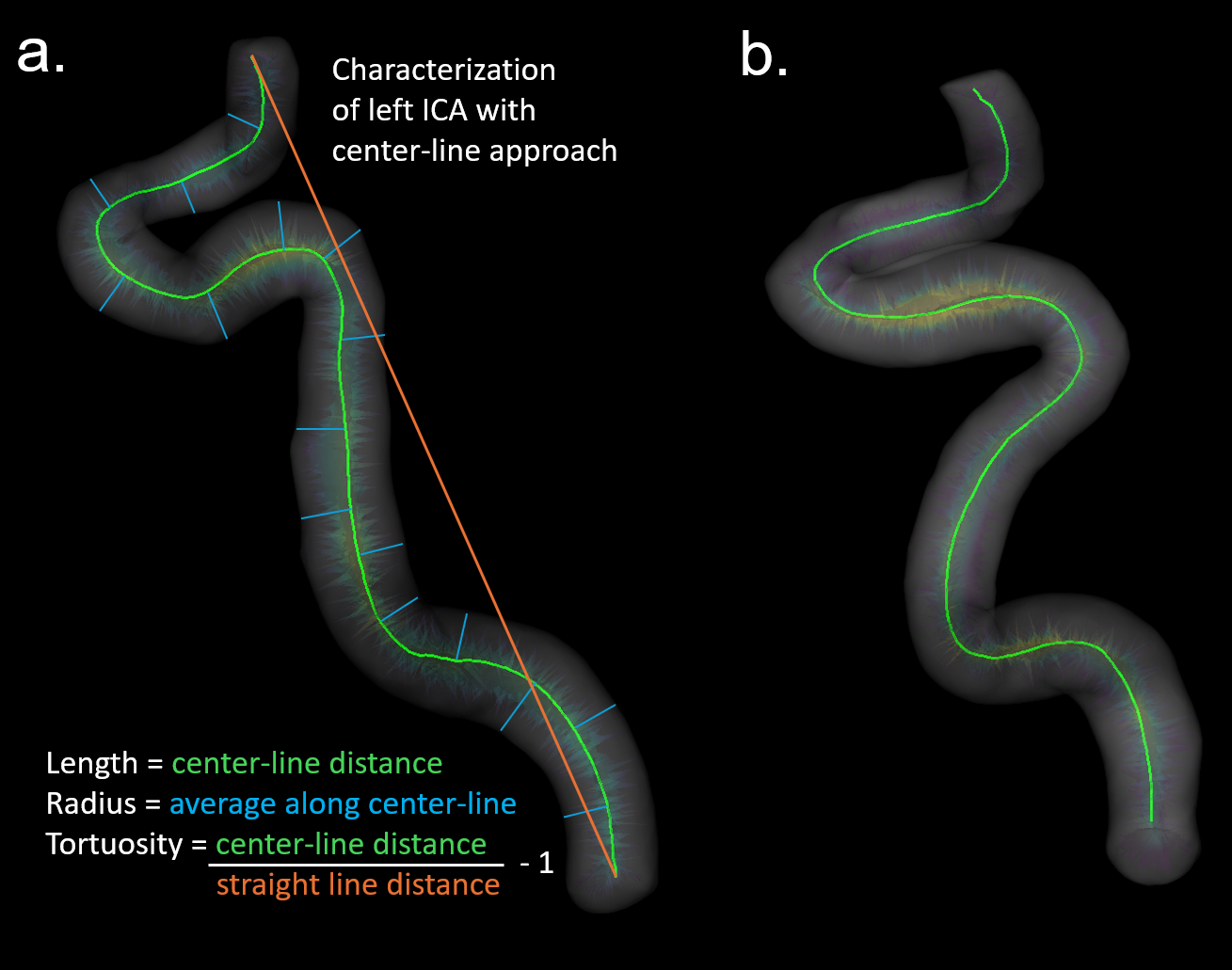

Supplement: Supplementary Data 4 — Calculation of centerline measures and comparison of ICA tortuosity between two individuals. a. Characterization of left ICA length, radius, and tortuosity. Left ICA tortuosity = 0.88. b. left ICA segment from second patient, tortuosity = 1.27. [file mmc4.zip › Supplementary_Figure_4.png]
