# Supplementary material for: Cerebrovascular longitudinal atlas: Changes in cerebral arteries in unruptured intracranial aneurysm patients followed with MRA
Source: Neuroimage Clin. 2025 Mar 7;46:103766. doi: 10.1016/j.nicl.2025.103766 (PMC11960659; doi:10.1016/j.nicl.2025.103766)
Supplement: Supplementary Data 5 [file mmc5.docx]

|  | Acceleration | Time Shift | Acceleration Factor | | Time Shift |  |  |  |  |
| --- | --- | --- | --- | --- | --- | --- | --- | --- | --- |
|  | p value | p value | 0 | 1 | 0 | 1 | 2 | 3 | 4 |
| Race | 0.242 | **0.022** |  |  | **47.229 (32.526)** | **40.269 (30.249)** | **56.493 (8.400)** | **39.028 (11.553)** | **67.759 (37.599)** |
| Ethnicity | 0.247 | **0.038** |  |  | **47.792 (29.114)** | **39.234 (30.696)** | **69.104 (42.195)** |  |  |
| Personal Hx of SAH | **0.023** | 0.463 | **0.453 (0.598)** | **0.168 (0.136)** |  |  |  |  |  |
| Hypertension | 0.169 | **0.0004** |  |  | **39.555 (32.804)** | **62.999 (32.229)** |  |  |  |
| Diabetes Mellitus | **0.016** | 0.405 | **0.418 (0.504)** | **0.622 (0.675)** |  |  |  |  |  |
| Cancer | 0.223 | **0.034** |  |  | **46.603 (30.750)** | **66.559 (38.432)** |  |  |  |
| Atherosclerosis/  ICA Calcification | 0.346 | **0.001** |  |  | **44.235 (34.151)** | **66.149 (30.527)** |  |  |  |
| ICA aneurysm | 0.126 | **0.013** |  |  | **66.247 (35.199)** | **46.572 (34.210)** |  |  |  |

**Supplementary Table 1. Median and interquartile range values for significant factors from longitudinal atlas results.** Categories 0 and 1 respectively represent “No” and “Yes” for corresponding variable. For Race, categories 0 – 4 are White, Asian, Black or African American, More than one race, and Unknown or Not reported, respectively. For Ethnicity, categories 0-2 are Not Hispanic or Latino, Hispanic or Latino, and Unknown, respectively. Plots are provided in Fig. 2 and Fig. 3.
