# Supplementary material for: Cerebrovascular longitudinal atlas: Changes in cerebral arteries in unruptured intracranial aneurysm patients followed with MRA
Source: Neuroimage Clin. 2025 Mar 7;46:103766. doi: 10.1016/j.nicl.2025.103766 (PMC11960659; doi:10.1016/j.nicl.2025.103766)
Supplement: Supplementary Data 6 [file mmc6.docx]

| Sex | Source 1 (0.019), Source 2 (0.005), Source 10 (0.008) |
| --- | --- |
| Race | Source 8 (0.004) |
| Multiple aneurysms | Source 10 (0.041) |
| Smoker | Source 7 (0.048), Source 9 (0.046) |
| ADPKD | Source 8 (0.0004) |
| Other surgery | Source 4 (0.036), Source 5 (0.045) |
| Stenosis of Carotid | Source 10 (0.041) |
| ICA aneurysm | Source 7 (0.0004) |
| MCA aneurysm | Source 2 (0.049), Source 6 (0.032) |
| Posterior aneurysm | Source 10 (0.018) |
| Aneurysm growth | Source 4 (0.020) |

**Supplementary Table 2. Significant geometric sources of variation for patient medical history and demographic factors.** Each source corresponds to a particular geometric source that differed between the groups. P-values for each significant source are provided in parentheses. Plots and illustrations of selected factors are provided in Figure 4.
